# New Prognostic Gene Signature and Immune Escape Mechanisms of Bladder Cancer

**DOI:** 10.3389/fcell.2022.775417

**Published:** 2022-05-12

**Authors:** Yi Jiang, Zhenhao Zeng, Situ Xiong, Ming Jiang, Gaomin Huang, Chiyu Zhang, Xiaoqing Xi

**Affiliations:** ^1^ Department of Urology, The Second Affiliated Hospital of Nanchang University, Nanchang, China; ^2^ Department of Urology, The First Affiliated Hospital of Nanchang University, Nanchang, China

**Keywords:** bladder cancer, immune microenvironment, gene signature, immune escape mechanism, prognosis

## Abstract

**Background:** The immune microenvironment profoundly affects tumor prognosis and therapy. The present study aimed to reveal potential immune escape mechanisms and construct a novel prognostic signature *via* systematic bioinformatic analysis of the bladder cancer (BLCA) immune microenvironment.

**Patients and Methods:** The transcriptomic data and clinicopathological information for patients with BLCA were obtained from The Cancer Genome Atlas (TCGA). Consensus clustering analysis based on the CIBERSORT and ESTIMATE algorithms was performed with patients with BLCA, which divided them into two clusters. Subsequently, the differentially expressed genes (DEGs) in the two were subjected to univariate Cox and least absolute shrinkage and selection operator (LASSO) regression analyses to identify prognostic genes, which were used to construct a prognostic model. The predictive performance of the model was verified by receiver operating characteristic (ROC) and Kaplan–Meier (K-M) analyses. In addition, we analyzed the differentially altered immune cells, mutation burden, neoantigen load, and subclonal genome fraction between the two clusters to reveal the immune escape mechanism.

**Results:** Based on the ESTIMATE and clustering analyses, patients with BLCA were classified into two heterogeneous clusters: ImmuneScoreH and ImmuneScoreL. Univariate Cox and LASSO regression analyses identified CD96 (HR = 0.83) and IBSP (HR = 1.09), which were used to construct a prognostic gene signature with significant predictive accuracy. Regarding potential immune escape mechanisms, ImmuneScoreH and ImmuneScoreL were characterized by inactivation of innate immune cell chemotaxis. In ImmuneScoreL, a low tumor antigen load might contribute to immune escape. ImmuneScoreH featured high expression of immune checkpoint molecules.

**Conclusion:** CD96 and IBSP were considered prognostic factors for BLCA. Innate immune inactivation and a low tumor antigen load may be associated with immune escape mechanisms in both clusters. Our research complements the exploration of the immune microenvironment in BLCA.

## Introduction

The most frequent tumor of the urogenital tract, bladder cancer (BLCA), is also one of the ten tumors with a notably poor prognosis (Kamat et al., 2016). Clinicopathological characteristics have been used to classify BLCA into two distinct categories: non-muscle-invasive bladder cancer (NMIBC) and muscle-invasive bladder cancer (MIBC). Most BLCA cases (∼60%) are NMIBC, which frequently recurs (50%–70%) but progresses slowly, with a 5-year survival rate of ∼90% (Prout et al., 1992). In contrast, patients with MIBC have a less favorable prognosis, with a 5-year survival rate of <50% (Knowles and Hurst, 2015). More importantly, at the time of initial diagnosis, approximately 5%–15% of patients with BLCA have metastatic disease (Kamat et al., 2013). Accurate predictions of tumor progression and the survival rate are essential for making appropriate diagnostic and treatment decisions. However, traditional methods are insufficient to accurately assess the prognosis of patients with BLCA. Therefore, it is essential to find effective biomarkers to evaluate and improve the diagnosis, treatment and prognosis of BLCA (Jordan and Meeks, 2019).

Notably, BLCA is considered immunogenic. Recently, increasing evidence suggests that the immune cell microenvironment is particularly important in BLCA. BLCA has long been considered a disease closely related to the immune system, and a variety of related immune cells and inflammatory biomarkers have been discovered (Joseph and Enting, 2019; Chen et al., 2020; Yejinpeng Wang et al., 2020). However, the immune cell microenvironment in BLCA has not been systematically evaluated. Therefore, in this study, the immune cell microenvironment of BLCA was studied, and the correlation of immune cell-related genes with the prognosis of patients with BLCA was systematically investigated.

Immune escape means that tumor cells escape immune surveillance by modifying their own surface antigens and remodeling the tumor microenvironment (TME); they then grow progressively, develop into uncontrollable tumors, and finally endanger the body (Crispen and Kusmartsev, 2020). Tumor immune escape involves a complex series of processes consisting of interactions between tumor cells and the immune system, and the mechanism is not completely clear. Immune monitoring, immune homeostasis and immune escape are three major processes involved in the occurrence and development of BLCA, and immune escape of cancer cells complicates the clinical treatment of BLCA. Increasing evidence indicates that apoptotic signal transduction from immune cells to BLCA cells is impaired, leading to immune evasion of BLCA cells. For example, binding of the antigen B7-H1 to PD-1 leads to functional inhibition of T and B cells, inhibition of body-specific cellular and humoral immunity, and induction of apoptosis in specific cytotoxic T lymphocytes (CTLs), all of which may explain the immune escape of BLCA cells and promote bladder tumor growth (Cancer Genome Atlas Research Network, 2014). However, the immune escape mechanism cannot be completely attributed to blockade of apoptotic signal transduction from immune cells to cancer cells—it is also regulated by a variety of genes. Thus, we further studied the regulation of immune escape genes in bladder tumors.

BLCA is an ideal disease model for studying immune escape. Previous reports indicate that immunosuppressive cells, including regulatory T cells, tumor macrophages and myeloid-derived suppressor cells, facilitate immune escape of BLCA. In addition, immune checkpoints regulate the different stages of the immune response and signal transduction processes to promote immune escape (Crispen and Kusmartsev, 2020; Jasinski-Bergner et al., 2022). However, the mechanism of immune escape in BLCA is incompletely understood.

Here, we integrated 414 BLCA samples from The Cancer Genome Atlas (TCGA), and after applying ESTIMATE, consensus clustering analysis, and differentially expressed gene (DEG) analysis, 2 immune-related genes (CD96 and IBSP) were extracted to construct a prognostic model for BLCA. In addition, the current results provide evidence indicating that innate immune inactivation and a low tumor antigen load are probably the primary immune escape mechanisms of BLCA, a finding that deserves further research.

## Materials and Methods

### Data Sources

The TCGA database consists of 414 tumor and 19 normal tissue samples, from which we obtained normalized RNA sequencing data and relevant clinical information available as of 24 September 2020. The normalized gene expression profiles were log2-transformed for subsequent analysis. Additionally, the GSE13507 dataset (https://www.ncbi.nlm.nih.gov/geo/query/acc.cgi?acc=GSE13507) containing 165 BLCA samples with complete survival information and clinical data for the corresponding patients was downloaded from the GEO database and considered an extrinsic validation set for testing the general applicability of the prognostic signature.

### Calculation of the Immune Cell Microenvironment Abundance

Newman et al. (2019) described a deconvolution algorithm, CIBERSORT, which is applied based on the normalized gene expression profile using LM22 files and 1,000 permutations to represent the cellular composition of a complex tissue. In the present study, CIBERSORT (http://cibersort.stanford.edu/) was used to assess the abundance of each of 22 infiltrating immune cell types in each sample. When CIBERSORT outputs a Monte Carlo *p* < 0.05 for a sample, the fraction of immune cell populations inferred by CIBERSORT for that sample is accurate and eligible for inclusion in the follow-up analysis (Newman et al., 2015). Based on this information, 178 patient samples were included in this analysis. The combined score of all immune cell types assessed was one for each sample. For comparison across different immune cell types and datasets, the cell fractions can be interpreted directly (Ciavarella et al., 2018).

### Consensus Clustering Analysis Based on Immune Infiltrating Cells

Consensus clustering analysis was performed using the ConsensusClusterPlus package. In brief, we typed 178 tumor samples with clinical information using the ConsensusClusterPlus package based on 22 infiltrating immune cell scores inferred by CIBERSORT, with the clustering method set to kmdist (clusterAlg = “kmdist”), the distance calculation method set to Euclidean (distance = “Euclidean”), and 1,000 repetitions to determine the accuracy of the classification. The StromalScore and ImmuneScore were also determined by the ESTIMATE algorithm based on transcriptome expression profiles of BLCA, and a clustering heatmap was generated. The analysis method was integrated into the “estimate” package in R (Yoshihara et al., 2013).

### Identification of Differentially Expressed Genes and Construction of the Prognostic Gene Signature

In this study, we used the “limma” package in R software to compare the differences in gene abundance between the two clusters (ImmuneScoreH vs. ImmuneScoreL). With |log2 fold change (FC)| > 1 and adjusted *p* < 0.05 as the threshold criteria to screen for DEGs, 22 DEGs were identified (Supplementary Table S1).

Here, a sample of 175 patients with BLCA for whom complete clinical information and survival data were available from the TCGA database was integrated as the training set. Similarly, the 165 BLCA samples in the GSE13507 dataset that met the aforementioned criteria were considered the extrinsic validation set. Univariate Cox regression analyses were conducted to identify the DEGs associated with overall survival (OS; *p* < 0.05). Subsequently, based on the “glmnet” package in R, least absolute shrinkage and selection operator (LASSO) logistic regression analyses were performed with 10-fold cross-validation to obtain the optimal variables. Ultimately, the variables identified by LASSO analysis were subjected to stepwise multivariate Cox regression analysis to determine the optimal variables for constructing a prognostic signature (*p* < 0.05; Supplementary Table S2). The formula for calculating the risk score is shown below.

The patients were classified into the high- and low-risk groups based on the median risk score. The median risk score for the TCGA-BLCA cohort was 0.9771; the median risk score for the GSE13507 dataset was −1.2357. The difference in survival between the two groups was evaluated by Kaplan–Meier (K-M) analysis. The area under the receiver operating characteristic (ROC) curve (AUC) was applied to assess the performance of the prognostic gene signature. In the extrinsic validation set, the same analyses (K-M survival analysis and ROC analysis) were performed to verify the validity of the prognostic signature.

### Analysis of Independent Prognostic Factors

Risk scores and clinicopathological characteristics (including age, sex, pathological stage, T stage, N stage, cluster, and ImmuneScore) were included in the univariate and multivariate Cox analyses to identify the independent prognostic factors for BLCA. *p* < 0.05 was considered statistically significant.

### Quantification of Tumor Mutation Burden

The TMB is considered the total number of somatic gene coding, base substitution, and gene insertion or deletion errors detected per million bases, and the TMB value has been used as a marker to determine a patient’s response to immune checkpoint inhibitor (ICI) treatment (Xu et al., 2019). We determined the TMB value for each sample using Perl scripts on the Java 8 platform.

### Selection of Neoantigens

After obtaining whole-exome sequencing (WES) data (.bam format) of paired normal samples from patients with BLCA, we first used the POLYSOLVER tool (Burstein et al., 2015) to infer the 4-digit HLA genotype for each sample. Then, neoantigens were predicted using NetMHCpan (v4.0) (Liu et al., 2016), with the somatic mutation data (.maf format) and HLA genotype data as the input. Neoantigens derived from the protein-coding single-nucleotide variants (SNVs) (Variant Classification = “Missense Mutation” and Variant Type = “SNP”) and small insertions and deletions (Indels) (Variant Classification = “Frame Shift_Ins,” “Frame Shift Del,” “In Frame Ins,” “In Frame Del” and Variant Type = “INS,” “DEL”) were predicted separately. Mutations that were predicted to produce peptides with an affinity < 500 nM and for which the corresponding gene was expressed at a level higher than the Combat value 1 (evaluated based on the median abundance rather than the abundance in a specific sample) were selected as neoantigens (Xiao et al., 2019).

### Estimation of Intratumoral Heterogeneity

ASCAT (Buchwald et al., 2020) was used to integrate copy number data with somatic mutation data to estimate the purity and ploidy of each tumor using default parameters. A modified PyClone workflow (Roth et al., 2014) was then used to estimate the cancer cell fractions in each sample. The fraction of cancer cell subclones was used as an indicator representing ITH (Xiao et al., 2019).

### Statistical Analysis

The Wilcoxon test and Kruskal–Wallis test were employed for analysis of mutation burden, neoantigen load, and ITH. Pearson correlation analysis was implemented to generate the correlation matrices. The K-M method with the log-rank test was used for survival analysis. Unless indicated otherwise, *p* < 0.05 was considered statistically significant. All analyses were performed using R software.

## Results

### Classification of Bladder Cancer Phenotypes Based on the Immune Microenvironment

**FIGURE 1:**
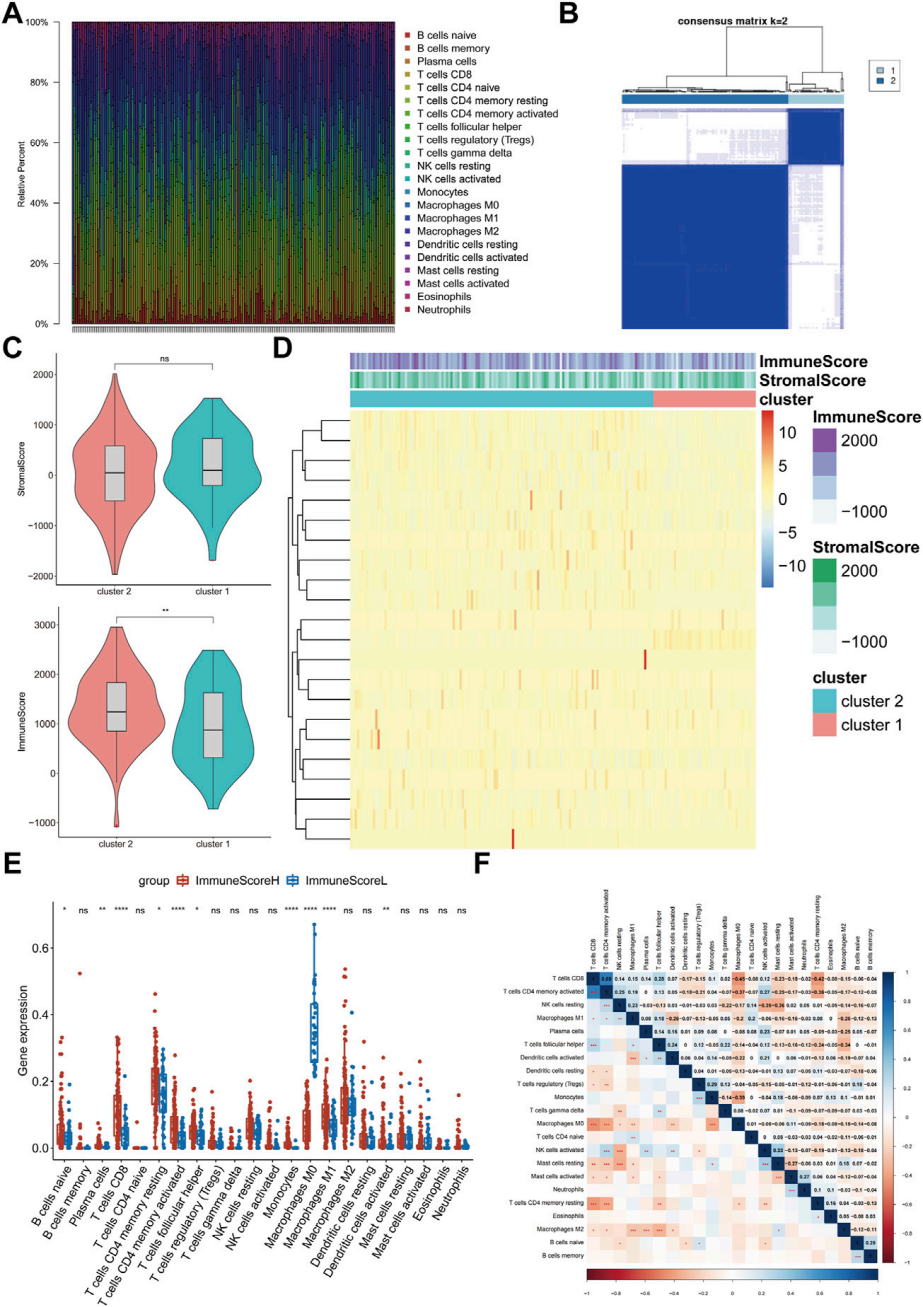
Evaluation and visualization of immune cell infiltration in BLCA. **(A)** Frequencies of immune cells in all samples. Stacked bar charts by sample name (*n* = 178). **(B)** Clustering heatmap showing the clusters at *k* = 2. **(C)** Differences between the immune scores of cluster 1 and cluster 2. **(D)** Abundances of infiltrating immune cell subsets based on ssGSEA between cluster 1 and cluster 2. **(E)** Differences in the infiltration levels of the 22 immune cell subsets between ImmuneScoreH and ImmuneScoreL. **(F)** Correlation matrix of all 22 immune cell frequencies. Some immune cells were negatively related (shown in blue), and others were positively related (shown in red). The darker the color, the higher the correlation was. *p < 0.05, **p < 0.01, ***p < 0.001, ****p < 0.0001, ns < 1.

We obtained 414 BLCA samples from the TCGA database. After filtering based on the criterion Monte Carlo *p* < 0.05, 178 samples were used for follow-up research. First, we assessed the abundance of each of 22 immune cell subsets in each sample to systematically present the state of the immune microenvironment in BLCA. Among the cell subsets, resting memory CD4^+^ T cells (average ratio = 0.18), M0 macrophages (average ratio = 0.14), and M2 macrophages (average ratio = 0.14) were exhibited the highest fractions (Figure 1A). Consequently, we then performed consensus clustering analysis of the BLCA samples (*n* = 408) based on the immune cell fractions (calculated with ssGSEA), and the patients were classified into two heterogeneous clusters (Figure 1B). Furthermore, the ESTIMATE algorithm identified extremely significant differences between the immune scores of the two different BLCA clusters (*p* < 0.01, Figure 1C).

The heatmap shows the abundances of immune cells and the distribution of the immune and stromal scores in the clusters. The immune score of Cluster 2 was relatively high; thus, Cluster 2 was named “ImmuneScoreH,” and Cluster 1 was designated “ImmuneScoreL” (Figure 1D). We compared the differences in the abundances of immune cells between the clusters. ImmuneScoreH contained mainly CD8^+^ T cells, activated memory CD4^+^ T cells, monocytes, and M1 macrophages (*p* < 0.0001). M0 macrophages were present at higher levels in ImmuneScoreL (*p* < 0.0001). The abundances of memory B cells, naive CD4^+^ T cells, NK cells, and mast cells did not differ significantly between the two clusters (Figure 1E). Additionally, we described the correlations between the immune cell subpopulations. CD8^+^ T cells were strongly positively correlated with activated memory CD4^+^ T cells (r = 0.69, *p* < 0.001), and M0 macrophages were negatively correlated with CD8^+^ T cells (r = −0.45, *p* < 0.001; Figure 1F). Notably, these 3 cell types had similar abundances, as shown in Figure 1E above.

### Prognostic Significance of Differentially Expressed Genes in Different Bladder Cancer Types

**FIGURE 2:**
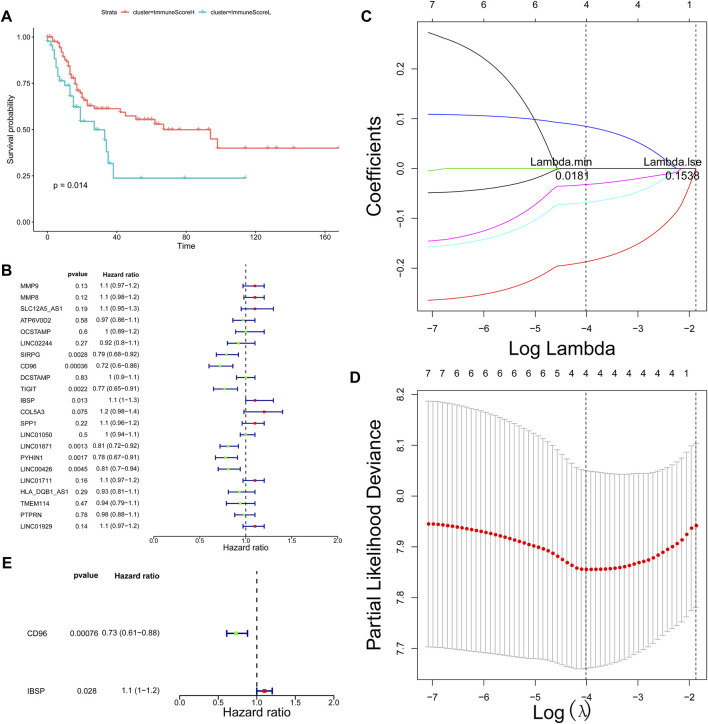
Screening of prognostic markers. **(A)** Survival analysis of patients in different ImmuneScore clusters. **(B)** Univariate Cox regression analysis showing the HR of each DEG in predicting OS in BLCA. **(C)** LASSO coefficient profiles of the 7 DEGs. **(D)** Tenfold cross‐validation for tuning parameter selection in the LASSO model. The red dots indicate the partial likelihood deviance values, the gray lines indicate the standard error (SE) values, and the two vertical dotted lines on the left and right indicate the optimal values determined by the minimum criteria and 1-SE criteria, respectively. “Lambda” is the tuning parameter. **(E)** Multivariate Cox analysis identified the optimal variables for constructing the prognostic signature in BLCA; *p* < 0.05.

**FIGURE 3:**
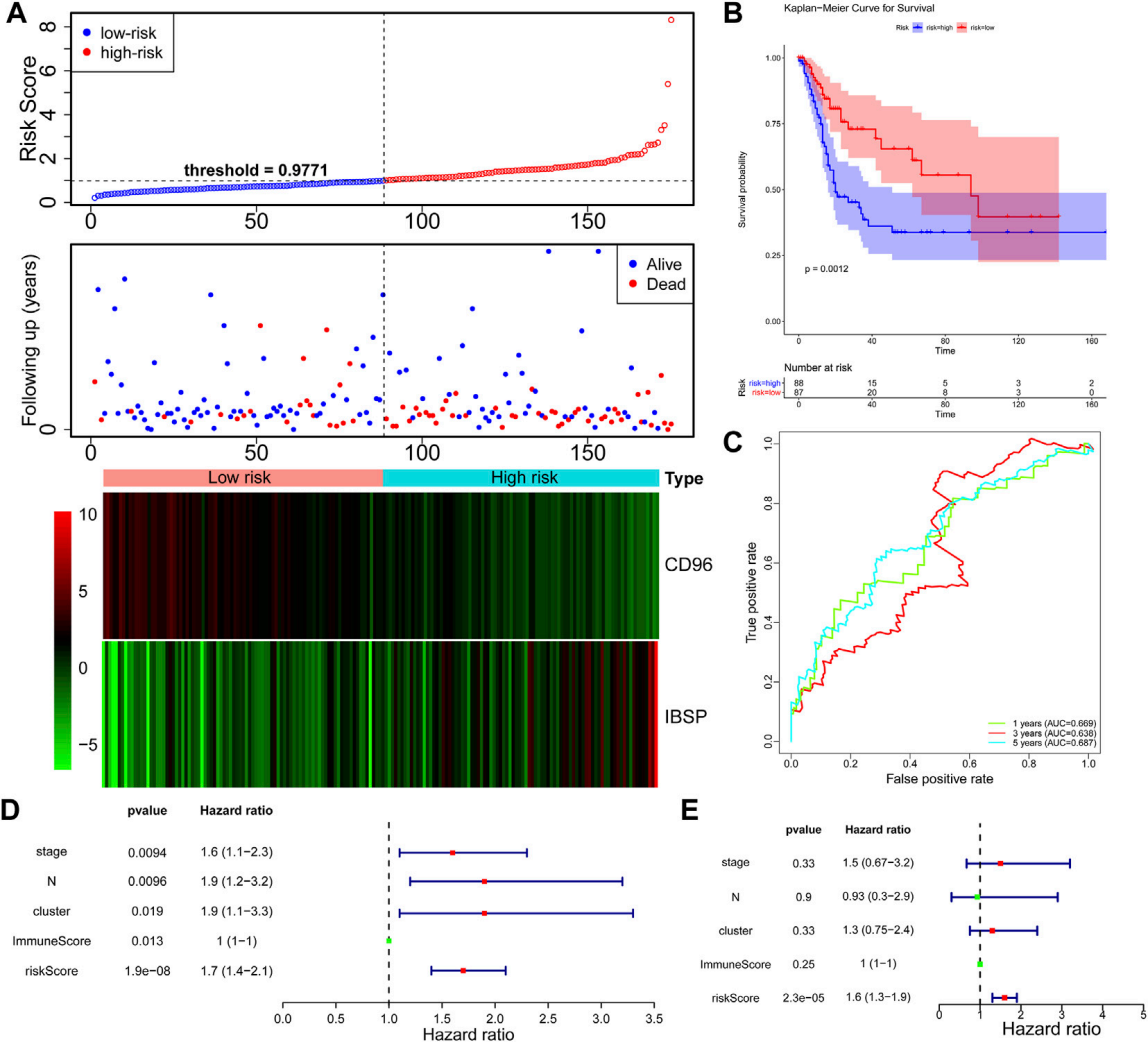
Construction of the DEG-based signature based on the TCGA dataset. **(A)** Distribution of the risk score, the associated survival data and heatmap of prognostic gene expression in the TCGA dataset. BLCA samples were divided into the high- and low-risk groups according to the median value of the risk score in the training set (threshold = 0.9771). **(B)** Survival analysis of patients in the high- and low-risk groups, which were established according to the abundance of the prognostic DEGs. **(C)** The AUC was used to measure the predictive accuracy of the 2-gene signature at 5 years (AUC = 0.817). **(D)** Univariate analysis was performed with the risk score and each clinical feature. **(E)** Subsequent multivariate Cox regression analysis showed that the risk score was the only independent risk factor for poor prognosis in BLCA patients.

Based on the important role of the immune cell microenvironment in determining prognosis (Zheng et al., 2020), we investigated the correlations between the immune score clusters and OS. ImmuneScoreH was associated with a significantly longer OS time (*p* = 0.014) than ImmuneScoreL (Figure 2A). However, the clinicopathological characteristics appeared to be unrelated to the clusters (Table 1). Then, we identified 22 DEGs in ImmuneScoreH and ImmuneScoreL. After univariate Cox analysis (Figure 2B; Supplementary Table S3), 7 genes—SIRPG [*p* = 0.0028, hazard ratio (HR) = 0.79, 95% CI = 0.8–0.92], CD96 (*p* = 0.00036, HR = 0.72, 95% CI = 0.6–0.86), TIGIT (*p* = 0.0022, HR = 0.77, 95% CI = 0.65–0.91), IBSP (*p* = 0.013, HR = 1.1, 95% CI = 1–1.3), LINC01871 (*p* = 0.0013, HR = 0.81, 95% CI = 0.72–0.92), PYHIN1 (*p* = 0.0017, HR = 0.78, 95% CI = 0.67–0.91), and LINC00426 (*p* = 0.0045, HR = 0.72, 95% CI = 0.6–0.86)—were included in LASSO Cox regression analysis. As shown in Figures 2C,D, in this analysis with lambda min = 0.0180997, 4 genes were identified (Supplementary Table S4): CD96 (HR = 0.83), IBSP (HR = 1.09), LINC01871 (HR = 0.93), and PYHIN1 (HR = 0.97). These genes were included in the subsequent multivariate Cox regression analysis. Ultimately, a prognostic signature based on CD96 and IBSP was established (Figure 2E). As the risk scores increased, the deaths clustered (Figure 3A, top). The K-M survival curves revealed a significant disadvantage in OS for patients in the high-risk group (*p* < 0.0001; Figure 3B). Additionally, the AUC of the time-dependent ROC curve indicated 1-, 3-, and 5-year prognostic accuracies of 0.669, 0.638, and 0.817, respectively, for the 2-gene signature (Figure 3C). The expression patterns of the two prognostic genes in the high- and low-risk groups are shown in the bottom panel of Figure 3A. We validated these results in the extrinsic validation set using the same analysis method. Consistent with the above results, the prognostic signature was applicable in the GSE13507 dataset. In brief, patients in the high-risk group were characterized by higher death rate and shorter OS times (Supplementary Figures S1A,B). Regarding the predictive validity of the prognostic signature, the AUCs for 1-, 3-, and 5-year survival in the extrinsic validation set were 0.712, 0.614, and 0.608, respectively (Supplementary Figure S1C). This evidence suggested that the prognostic signature based on CD96 and IBSP was applicable to most patients with BLCA and had reliable predictive prognostic validity.

**TABLE 1:** The Clinicopathologic characteristics of the immune score clusters.

Cluster				
	Total (*N* = 175)	ImmuneSacoreH (*N* = 131)	ImmuneScoreL (*N* = 44)	*p*-Value
Gender				
Female	52 (29.7%)	35 (26.7%)	17 (38.6%)	0.192
Male	123 (70.3%)	96 (73.3%)	27 (61.4%)	
Age (years)				
≥60	27 (15.4%)	24 (18.3%)	3 (6.8%)	0.113
<60	148 (84.6%)	107 (81.7%)	41 (93.2%)	
Histologic grade				
High grade	173 (98.9%)	129 (98.5%)	44 (100%)	1
Low grade	1 (0.6%)	1 (0.8%)	0 (0%)	
Missing	1 (0.6%)	1 (0.8%)	0 (0%)	
T stage				
T1	1 (0.6%)	1 (0.8%)	0 (0%)	0.742
T2	41 (23.4%)	31 (23.7%)	10 (22.7%)	
T3	102 (58.3%)	73 (55.7%)	29 (65.9%)	
T4	21 (12.0%)	17 (13.0%)	4 (9.1%)	
Missing	10 (5.7%)	9 (6.9%)	1 (2.3%)	
N stage				
N0	104 (59.4%)	83 (63.4%)	21 (47.7%)	0.222
N1	26 (14.9%)	18 (13.7%)	8 (18.2%)	
N2	29 (16.6%)	18 (13.7%)	11 (25.0%)	
N3	3 (1.7%)	2 (1.5%)	1 (2.3%)	
Missing	13 (7.4%)	10 (7.6%)	3 (6.8%)	
M stage				
M0	72 (41.1%)	58 (44.3%)	14 (31.8%)	0.866
M1	2 (1.1%)	1 (0.8%)	1 (2.3%)	
Missing	101 (57.7%)	72 (55.0%)	29 (65.9%)	

**TABLE 2:** Independent prognostic value of risk score in multivariate Cox analysis.

	Coefficient	HR	Z	*p*-Value
Age	−0.71	0.49 (0.23–1)	−1.9	0.063
Stage	0.19	1.2 (0.53–2.7)	0.44	0.66
N	0.34	1.4 (0.44–4.5)	0.57	0.57
riskScore	0.36	1.4 (1.3–1.6)	5.4	7.10E-08

Furthermore, we explored the independent prognostic value of the 2-gene signature. We included age, sex, pathological stage, T stage, N stage, cluster, ImmuneScore, and risk score in the univariate Cox regression analysis and found that T stage (*p* = 0.0094), N stage (*p* = 0.0096), cluster (*p* = 0.019), ImmuneScore (*p* = 0.013), and risk score (*p* = 1.9e-08) were candidate prognostic factors (Figure 3D). However, subsequent multivariate Cox regression analysis showed that the risk score was the only independent risk factor for poor prognosis in patients with BLCA (Figure 3E; Table 2).

### Potential Extrinsic Immune Escape Mechanisms of Bladder Cancer

Based on the heterogeneity in the BLCA immune cell phenotypes, we speculated that each BLCA cluster might have different mechanisms of tumor immune escape. We first investigated the extrinsic mechanisms of immune escape (Schreiber et al., 2011), which comprise four main dimensions: lack of immune cells, presence of immunosuppressive cells (e.g., M2 macrophages and regulatory T cells), high levels of immunosuppressive cytokines (e.g., IL-10 and TGF-β), and fibrosis (Spranger, 2016).

**FIGURE 4:**
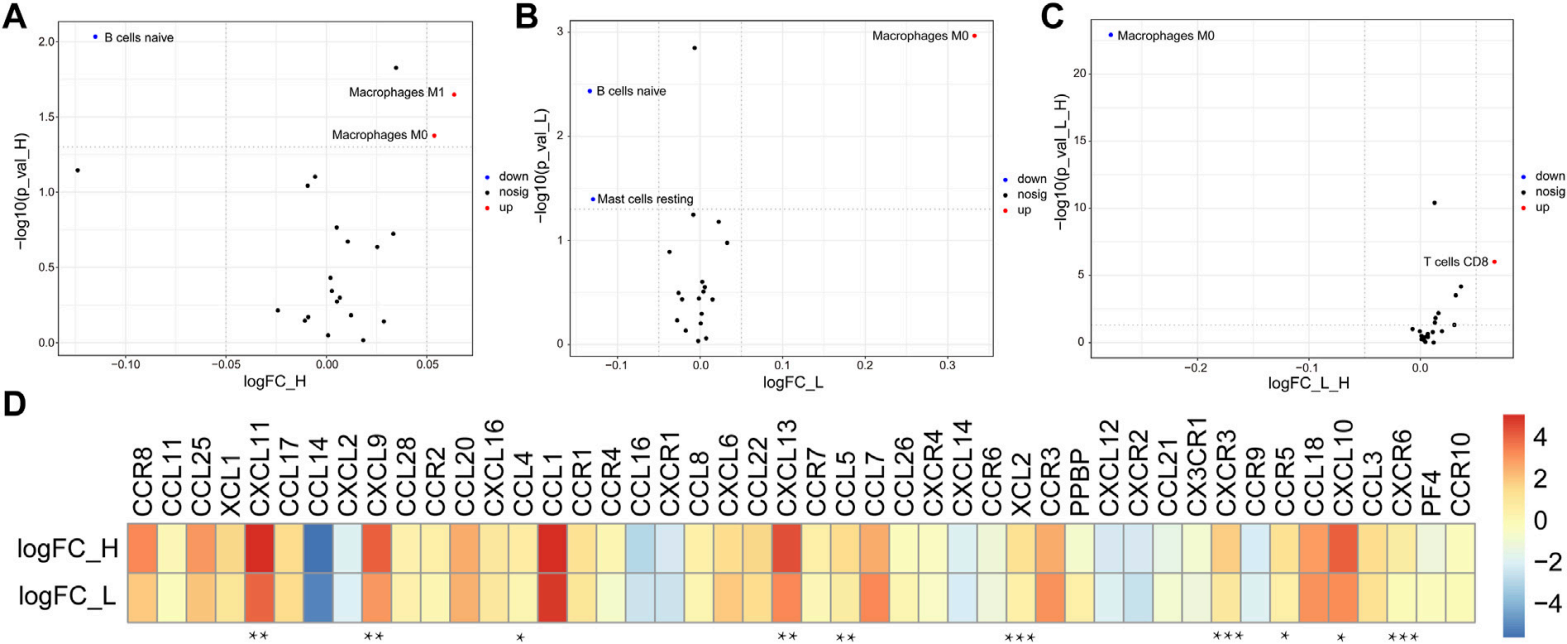
Potential extrinsic immune escape mechanisms of BLCA. **(A,B)** Volcano plots of more abundant (red) and less abundant (blue) immune cells in tumor samples compared with the paired normal samples in each group. **(C)** The differentially abundant immune cells between ImmuneScoreH and the ImmuneScoreL are shown in a volcano plot. **(D)** Log2 ratio of chemokines in each cluster. Molecules with significant differential expression between ImmuneScoreH and the ImmuneScoreL (*p* < 0.05) are shown. *p < 0.05, **p < 0.01, ***p < 0.001.

Compared with the normal group, the results showed that the abundance of M0 and M1 macrophages were significantly increased in ImmuneScoreH, but naïve B cells were decreased (Figure 4A). The trends in the abundance of M0 macrophages and naïve B cells in ImmuneScoreL compared to the normal group were consistent with their abundance in ImmuneScoreH, and the number of resting mast cells was significantly reduced (Figure 4B). In addition, we focused on the relationship between resting memory CD4^+^ T cells, M0 macrophages, and M2 macrophages and patient prognosis. However, these immune cell subsets did not seem to be directly related to clinical outcomes in BLCA patients (all *p* > 0.05, Supplementary Figure S2). Furthermore, we found a significant positive correlation between M0 macrophages and risk score (cor = 0.458892, *p* = 1.18E-10; Supplementary Table S5). Compared to ImmuneScoreL, M0 macrophages were reduced in ImmuneScoreH and T cells CD8 were increased (Figure 4C). Taken together, the enrichment of inactive M0 macrophages in both clusters may impair anti-tumor immune response through the immune system, thus representing a potential extrinsic mechanism of immune escape.

Figure 4D showed that the abundance of most chemokines were high in both groups. Specifically, CXCL11, CXCL9, CCL4, CXCL13, CCL5, XCL2, CXCR3, CCR5, CXCL10, and CXCR6 were expressed at significantly higher levels in ImmuneScoreH than in the ImmuneScoreL group (all *p* < 0.05). In addition, we focused on the correlation of the risk score with the abundance of 10 differentially expressed chemokines (CXCR6, CXCR3, CCR5, CXCL9, XCL2, CCL5, CXCL13, CCL4, CXCL10, and CXCL11) in the ImmuneScoreH and ImmuneScoreL groups. The risk scores were negatively correlated with the abundance of all 10 chemokines listed above. The strongest correlation was observed for CXCR6 (cor = −0.5087, *p* = 4.35E-12), followed by CXCR3 (cor = −0.45561, *p* = 1.89E-09) and CCR5 (cor = −0.4294, *p* = 2.19E-08) (Supplementary Figure S3A). Subsequently, the abundance of seven chemokines with |cor| > 0.3 and *p* < 0.05 (Supplementary Table S6) was further evaluated by K-M survival analysis of OS for patients with BLCA. High expression of each chemokine except XCL2 (i.e., CXCR6, CXCR3, CCR5, CXCL9, CCL5, and CXCL13) was significantly associated with better prognosis in patients with BLCA (all *p* < 0.05, Supplementary Figure S3B).

### Tumor Immunogenicity in Bladder Cancer

Here, we focused primarily on tumor immunogenicity and the abundance of antigen-presenting molecules and immune checkpoint molecules to characterize the potential intrinsic immune escape mechanism of BLCA (Schreiber et al., 2011).

**FIGURE 5:**
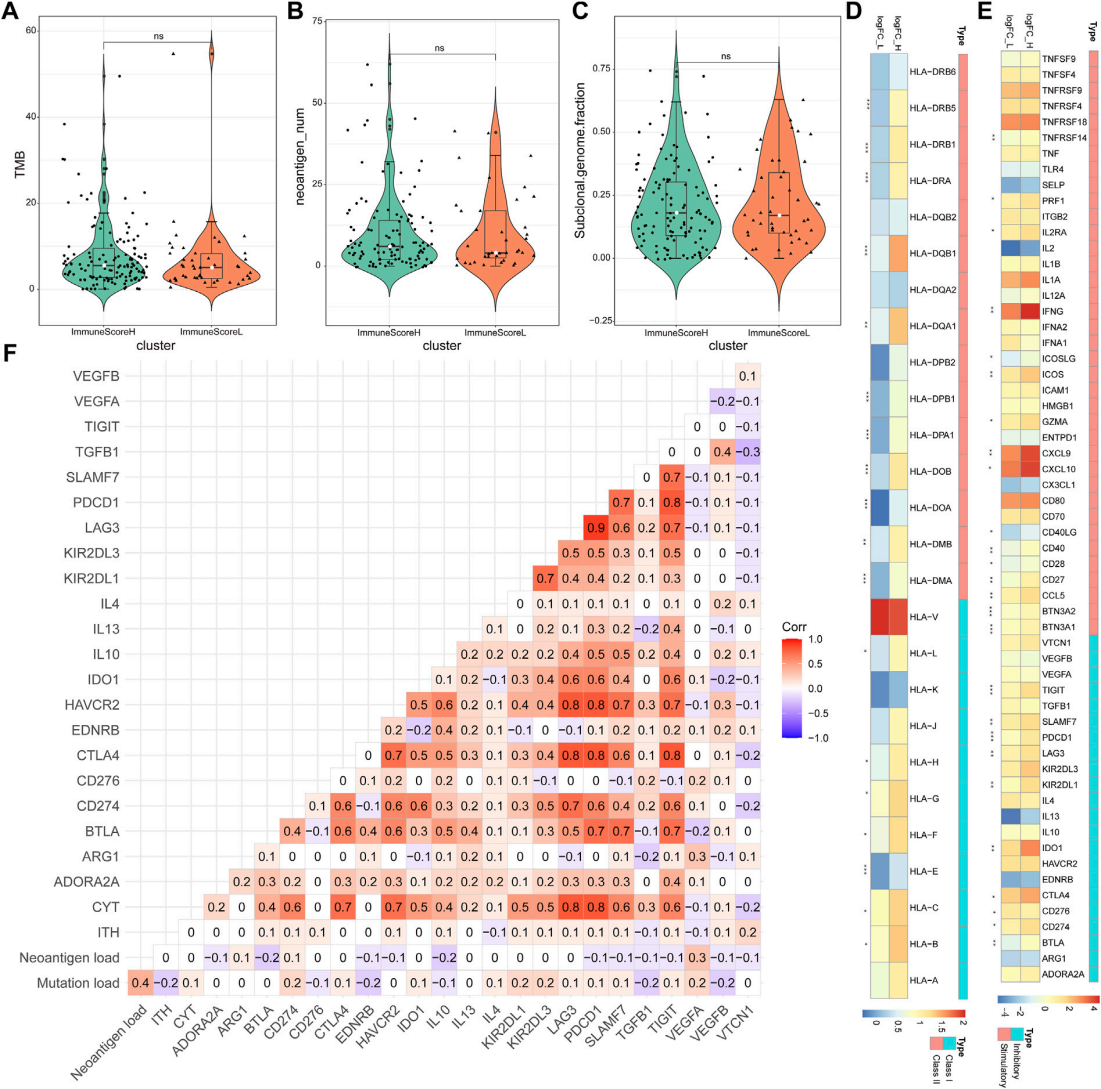
Potential intrinsic immune escape mechanisms of BLCA. Comparison of TMB **(A)**, neoantigen load **(B)**, and ITH **(C)** between the two clusters. In the violin plots, the mean values are plotted as white dots, and the boxplot was drawn inside the violin plot. **(D)** Comparison of the log2 FCs in HLA molecule expression in ImmuneScoreH compared with ImmuneScoreL. **(E)** Significant differential expression of costimulatory and coinhibitory molecules between the two clusters (*p* < 0.01). *p < 0.05, **p < 0.01, ***p < 0.001. **(F)** Correlations between tumor immunogenicity indicators and the expression of immune checkpoint molecules.

First, the potential factors that determined differences in tumor immunogenicity between the two clusters, including TMB (Figure 5A), neoantigen load (Figure 5B), and ITH (as the subclonal genome fraction; Figure 5C), were compared. Despite the lack of significant differences in the aforementioned factors between the two groups, ImmuneScoreL still exhibited a trend toward a reduced tumor antigen load. Moreover, antigen-presenting molecule abundance was lower in ImmuneScoreL than in ImmuneScoreH, contributing to the low immunogenicity in ImmuneScoreL (Figure 5D). In conclusion, no major difference in tumor immunogenicity was observed between the two clusters, but ImmuneScoreL was slightly less immunogenic.

Furthermore, Pearson correlation analysis showed that the risk score was significantly negatively correlated (|cor| > 0.3 and *p* < 0.05; Supplementary Table S7) with 9 of the 18 antigen-presenting molecules differentially expressed between ImmuneScoreH and ImmuneScoreL (HLA-DOB, HLA-DOA, HLA-DMA, HLA-DRA, HLA-DMB, HLA-DQA1, HLA-DPA1, HLA-DQB1, and HLA-DRB5), with HLA-DOB showing the strongest negative correlation with the risk score (cor = −0.41231, *p* = 2.34E-07). However, survival analysis suggested that the abundance of these nine antigen-presenting molecules was not associated with the prognosis of patients with BLCA (Supplementary Figure S4).

### Regulation of Immunomodulators in Bladder Cancer

Another essential mechanism of intrinsic immune escape is the abundance of immune checkpoint molecules following immune stimulation (Marin-Acevedo et al., 2018). We therefore compared the expression patterns of multiple immunomodulators (costimulatory and coinhibitory molecules; from https://www.rndsystems.com/cn/search?keywords=costimulatory-and-coinhibitory-molecules) between the clusters (Zou and Chen, 2008). ImmuneScoreH exhibited higher abundance of costimulatory and immune checkpoint molecules (most *p* < 0.05; Figure 5E), which may be a key mechanism by which tumors in the ImmuneScoreH cluster underwent immune-mediated killing after immune stimulation. Furthermore, Pearson correlation analysis showed that cytolytic activity (CYT) was positively correlated with the abundance of most checkpoint molecules, whereas mutation burden, neoantigen load, and ITH appeared to be independent of these molecules (Figure 5F). The risk score was significantly negatively correlated (|cor| > 0.3 and *p* < 0.05; Supplementary Table S8) with 15 of the 26 immunomodulators differentially expressed between ImmuneScoreH and ImmuneScoreL (TIGIT.x, CD27, PDCD1, CD40LG, ICOS, SLAMF7, CTLA4, CD28, BTLA, BTN3A1, CXCL9, CCL5, IL2RA, PRF1, and LAG3), with TIGIT.x showing the strongest negative correlation with the risk score (cor = −0.55456, *p* = 3.64E-14). Survival analysis showed that low abundance of 12 of these 15 immunomodulators (all except for CD27, CD28, and CD40LG) was associated with poor prognosis in patients with BLCA (Supplementary Figure S5).

## Discussion

In this study, a BLCA prognostic model including CD96 and IBSP was constructed, and the immune escape mechanisms of BLCA with different immune score classifications were investigated on the basis of ESTIMATE and consensus clustering analyses.

The TME is complex and constantly evolving (Hinshaw and Shevde, 2019). In addition to stromal cells, fibroblasts and endothelial cells, the TME also contains innate and adaptive immune cells (Wels et al., 2008; Hanahan and Weinberg, 2011). Studies conducted in the past two decades have shown that the TME is a determinant of tumor behavior equally important to the tumor cells themselves (Vitale et al., 2019). In the present study, BLCA exhibited two TME phenotypes. ImmuneScoreH contained mainly CD8^+^ T cells, CD4^+^ T cells, monocyte–macrophages (M1 and M2), and dendritic cells (DCs) (*p* < 0.05). The abundance of M0 macrophages was significantly higher in ImmuneScoreL (*p* < 0.0001). The simplest distinction between T cells is their division into the CD4^+^ and CD8^+^ subsets (van der Leun et al., 2020). CD8^+^ T cells play a crucial role in mediating antitumor immunity, and their effector cells—CD8^+^ CTLs—recognize tumor-associated antigens presented by major histocompatibility complex I molecules by expressing T-cell receptors to clear tumor cells (Fu and Jiang, 2018). Although CD8^+^ T cells are considered to be the main driving force of antitumor immunity, CD4^+^ T cells also play an important role in tumor control. For example, the anti-tumor immunity is mainly mediated by T cells, which have immune memory function and specific killing effect. CD4 and CD8 are two subgroups of T cells, which play a key role in the antigen presentation of dendritic cells (DC). CD4^+^ T cells can recognize antigens homologous with CD8^+^ T cells, activate antigen presenting cells, and activate CD8^+^ T cells, enhance their migration ability, the abundance of costimulatory molecules and the production of inflammatory cytokines (CTLs). CD4^+^ T cells help initiate the gene expression program of CD8^+^ T cells, enhance the function of CTLs by a variety of molecular mechanisms, and exert anti-tumor immunity (Borst et al., 2018; Balança et al., 2021). Thus, CD4^+^ and CD8^+^ T cells are the core of anti-tumor immunity research. In many human malignancies, the presence of T cells is associated with better prognosis in patients (van der Leun et al., 2020). In addition, studies have shown that a high level of macrophage infiltration in most tumor types (including breast, gastric, lung, liver, and other malignant tumors) is associated with poor prognosis (Cardoso et al., 2014; Zhao et al., 2017). Notably, our study also showed that the patients in ImmuneScoreH, with high levels of CD4^+^/8^+^ T cells, had excellent OS. Although the prognosis of the patients in ImmuneScoreL was poor, further studies are needed to confirm whether macrophages were a causal factor.

Considering the lack of prognostic biomarkers for BLCA and the findings described above, we applied univariate and LASSO Cox regression analyses based on the TCGA-BLCA dataset to identify the DEGs (ImmuneScoreH vs. ImmuneScoreL) related to OS and constructed a 4-gene prognostic signature. CD96, a new checkpoint receptor for cancer immunotherapy, may be a targeted receptor in T-cell and NK-cell biology for improving the immune response (Dougall et al., 2017). In liver cancer, high expression of CD96 is closely associated with deterioration and shorter disease-free survival (DFS) and OS times in patients, and it is accompanied by a low efficacy of antitumor immunotherapy (Sun et al., 2019). On the other hand, CD96 expression has not been reported to be high in BLCA. However, this study showed a significant association of high CD96 expression with poor prognosis in BLCA.

IBSP is a member of the n-linked glycoprotein family of integrin-binding ligands, which also includes osteopontin, dental matrix protein, salivary phosphoprotein and extracellular phosphorylated glycoproteins. Many studies have indicated that most members of this protein family are expressed mainly in bone tissues, although abnormal expression of some proteins is also found in malignant tumor tissues. Abnormal expression of the IBSP gene is closely associated with bone metastasis, an increased risk of malignancy and poor prognosis in patients with breast cancer, prostate cancer and non-small-cell lung cancer (Gordon et al., 2009; Wang et al., 2019; Wu et al., 2021). Unexpectedly, our study showed that IBSP was overexpressed in high-risk patients with BLCA, further indicating that abnormal expression of this gene is closely related to malignant tumors.

The tumor cell microenvironment is closely associated with the aggregation of a variety of immune cells with immunosuppressive phenotypes, such as bone marrow-derived suppressor cells (MDSCs), tolerogenic dendritic cells (tDCs), tumor-associated macrophages (TAMs) and regulatory T cells (Kaneda et al., 2017). Previous research has shown that high levels of prostaglandin E2 inhibit the differentiation of DCs, promote the accumulation of MDSCs, and drive the abundance of cytokines to promote the expansion of DCs, M2 macrophages and Th2 cells (Remes Lenicov et al., 2018). We speculated that prostaglandins might play an important role in this process. In addition, CCR2/CCL2, CCR8/CCL1 and CCL18 are newly identified targets that alter the immunosuppressive microenvironment and improve the effect of immunotherapy in BLCA (Tao Wang et al., 2020; Tu et al., 2020). This study identified enrichment of CCL4, CXCL9 and CXCL10 in ImmuneScoreH, suggesting the potentially important roles of these chemokines, although the specific mechanism underlying these roles remains to be investigated.

Although these genes have been verified in the TCGA database, multiomics data (such as mutation, copy number variation, and DNA methylation data) must be integrated for further analysis and validation. In addition, due to the insufficient number of clinical samples, experimental verification has not yet been performed. However, we will conduct follow-up experiments with a sufficient sample size in the future to verify the results of this research at the cellular and molecular levels.

In summary, several bioinformatics methods were used to identify TME subtypes in BLCA, and we identified a series of prognostic genes related to the tumor cell microenvironment. Moreover, this study suggests a potential novel immune escape mechanism of BLCA.

## Data Availability

The datasets presented in this study can be found in online repositories. The names of the repository/repositories and accession number(s) can be found in the article/Supplementary Material, further inquiries can be directed to the corresponding authors.
