# Supplementary material for: New Prognostic Gene Signature and Immune Escape Mechanisms of Bladder Cancer
Source: Front Cell Dev Biol. 2022 May 12;10:775417. doi: 10.3389/fcell.2022.775417 (PMC9133907; doi:10.3389/fcell.2022.775417)
Supplement: Supplementary file 1 [file Table1.DOCX]

| Supplementary Table S1: The total of 22 genes were screened from ImmuneScoreH vs. ImmuneScoreL. | |
| --- | --- |
| MMP9 |  |
| MMP8 |  |
| SLC12A5_AS1 |  |
| ATP6V0D2 |  |
| OCSTAMP |  |
| LINC02244 |  |
| SIRPG |  |
| CD96 |  |
| DCSTAMP |  |
| TIGIT |  |
| IBSP |  |
| COL5A3 |  |
| SPP1 |  |
| LINC01050 |  |
| LINC01871 |  |
| PYHIN1 |  |
| LINC00426 |  |
| LINC01711 |  |
| HLA_DQB1_AS1 |  |
| TMEM114 |  |
| PTPRN |  |
| LINC01929 |  |

Supplementary Table S2: The best prognostic genes identified by the multivariate Cox analysis.

|  | Coefficient | HR | z | p.value |
| --- | --- | --- | --- | --- |
| CD96 | -0.23 | 0.8 (0.64-0.99) | -2 | 0.041 |
| IBSP | 0.11 | 1.1 (1-1.2) | 2.1 | 0.039 |
| LINC01871 | -0.1 | 0.9 (0.74-1.1) | -1 | 0.31 |
| PYHIN1 | -0.024 | 0.98 (0.75-1.3) | -0.18 | 0.86 |

HR: Hazard Ratio.

Supplementary Table S3: The result of 7 genes was analyzed by the univariate Cox regression.

|  | Coefficient | HR (95% CI for HR) | wald.test | z | p.value |
| --- | --- | --- | --- | --- | --- |
| SIRPG | -0.23 | 0.79 (0.68-0.92) | 9 | -3 | 0.0028 |
| CD96 | -0.32 | 0.72 (0.6-0.86) | 13 | -3.6 | 0.00036 |
| TIGIT | -0.26 | 0.77 (0.65-0.91) | 9.3 | -3.1 | 0.0022 |
| IBSP | 0.14 | 1.1 (1-1.3) | 6.2 | 2.5 | 0.013 |
| LINC01871 | -0.21 | 0.81 (0.72-0.92) | 10 | -3.2 | 0.0013 |
| PYHIN1 | -0.25 | 0.78 (0.67-0.91) | 9.8 | -3.1 | 0.0017 |
| LINC00426 | -0.21 | 0.81 (0.7-0.94) | 8.1 | -2.8 | 0.0045 |

HR: Hazard Ratio, CI: confidence interval.

Supplementary Table S4: The results of LASSO regression analysis.

| Gene | Coefficient | HR |
| --- | --- | --- |
| CD96 | -0.187116431 | 0.829347169 |
| IBSP | 0.084622408 | 1.088306054 |
| LINC01871 | -0.06812555 | 0.934143185 |
| PYHIN1 | -0.032163944 | 0.968347814 |

HR: Hazard Ratio.

Supplementary Table S5: The Pearson correlation analysis between risk score and immune-infiltrating cells.

| Immune_cells | Riskscore | Correlation | P |
| --- | --- | --- | --- |
| Macrophages M0 | riskScore | 0.458892 | 1.18E-10 |
| Dendritic cells resting | riskScore | 0.23268 | 0.001775 |
| T cells gamma delta | riskScore | 0.202163 | 0.006806 |
| Mast cells activated | riskScore | 0.088696 | 0.239063 |
| T cells CD4 naive | riskScore | 0.07516 | 0.318715 |
| Macrophages M2 | riskScore | 0.064477 | 0.392515 |
| Neutrophils | riskScore | 0.03519 | 0.640978 |
| NK cells activated | riskScore | 0.010747 | 0.886778 |
| Eosinophils | riskScore | 0.004373 | 0.953801 |
| Mast cells resting | riskScore | -0.00456 | 0.951788 |
| T cells CD4 memory resting | riskScore | -0.01147 | 0.87925 |
| Dendritic cells activated | riskScore | -0.02127 | 0.778054 |
| Plasma cells | riskScore | -0.06871 | 0.362104 |
| B cells memory | riskScore | -0.07344 | 0.32992 |
| T cells follicular helper | riskScore | -0.11899 | 0.113666 |
| Macrophages M1 | riskScore | -0.12124 | 0.106935 |
| NK cells resting | riskScore | -0.12497 | 0.096492 |
| Monocytes | riskScore | -0.15011 | 0.045509 |
| T cells regulatory (Tregs) | riskScore | -0.19937 | 0.007629 |
| B cells naive | riskScore | -0.24891 | 0.000807 |
| T cells CD4 memory activated | riskScore | -0.29057 | 8.33E-05 |
| T cells CD8 | riskScore | -0.37836 | 1.92E-07 |

Supplementary Table S6: The result of Pearson correlation analysis between risk scores and Chemokines.

|  | Correlation | pvalue |
| --- | --- | --- |
| CXCR6 | -0.51087 | 4.35E-12 |
| CXCR3 | -0.45561 | 1.89E-09 |
| CCR5 | -0.4294 | 2.19E-08 |
| CXCL9 | -0.35227 | 1.14E-05 |
| XCL2 | -0.34261 | 1.95E-05 |
| CCL5 | -0.34251 | 1.95E-05 |
| CXCL13 | -0.31916 | 6.32E-05 |
| CCL4 | -0.27035 | 0.000854 |
| CXCL10 | -0.25332 | 0.001387 |
| CXCL11 | -0.2452 | 0.001387 |

Supplementary Table S7: The result of Pearson correlation analysis between risk score and differentially expressed antigen-presenting molecules.

|  | correlation | pvalue |
| --- | --- | --- |
| HLA-DOB | -0.41231 | 2.34E-07 |
| HLA-DOA | -0.36843 | 8.29E-06 |
| HLA-DMA | -0.35258 | 2.55E-05 |
| HLA-DRA | -0.34211 | 5.04E-05 |
| HLA-DMB | -0.33897 | 5.86E-05 |
| HLA-DQA1 | -0.33316 | 8.11E-05 |
| HLA-DPA1 | -0.32089 | 0.000169 |
| HLA-DQB1 | -0.31601 | 0.000213 |
| HLA-DRB5 | -0.30124 | 0.000485 |
| HLA-DPB1 | -0.29349 | 0.000694 |
| HLA-DRB1 | -0.28874 | 0.000814 |
| HLA-E | -0.27704 | 0.001382 |
| HLA-C | -0.23219 | 0.011578 |
| HLA-B | -0.22088 | 0.0161 |
| HLA-L | -0.22063 | 0.0161 |
| HLA-F | -0.21969 | 0.0161 |
| HLA-H | -0.16071 | 0.066214 |
| HLA-G | -0.13352 | 0.077297 |

Supplementary Table S8: The result of Pearson correlation analysis between risk score and differentially expressed immunomodulators.

|  | correlation | P value |
| --- | --- | --- |
| TIGIT.x | -0.55456 | 3.64E-14 |
| CD27 | -0.50708 | 1.72E-11 |
| PDCD1 | -0.45972 | 3.31E-09 |
| CD40LG | -0.42216 | 1.23E-07 |
| ICOS | -0.40804 | 4.17E-07 |
| SLAMF7 | -0.39841 | 9.12E-07 |
| CTLA4 | -0.39817 | 9.12E-07 |
| CD28 | -0.38709 | 2.12E-06 |
| BTLA | -0.37981 | 3.61E-06 |
| BTN3A1 | -0.36065 | 1.49E-05 |
| CXCL9 | -0.35227 | 2.61E-05 |
| CCL5 | -0.34251 | 4.90E-05 |
| IL2RA | -0.32873 | 0.000118 |
| PRF1 | -0.32455 | 0.000144 |
| LAG3 | -0.30779 | 0.000389 |
| IFNG | -0.29087 | 0.000989 |
| TNFRSF14 | -0.27051 | 0.002823 |
| IDO1 | -0.25856 | 0.004774 |
| CXCL10 | -0.25332 | 0.005546 |
| BTN3A2 | -0.22886 | 0.015745 |
| KIR2DL1 | -0.22379 | 0.015745 |
| CD274 | -0.21148 | 0.019361 |
| CD40 | -0.20541 | 0.019361 |
| GZMA | -0.12552 | 0.193884 |
| ICOSLG | -0.11429 | 0.193884 |
| CD276 | 0.225771 | 0.015745 |
