# Supplementary figures and images for: New Prognostic Gene Signature and Immune Escape Mechanisms of Bladder Cancer
Source: Front Cell Dev Biol. 2022 May 12;10:775417. doi: 10.3389/fcell.2022.775417 (PMC9133907; doi:10.3389/fcell.2022.775417)

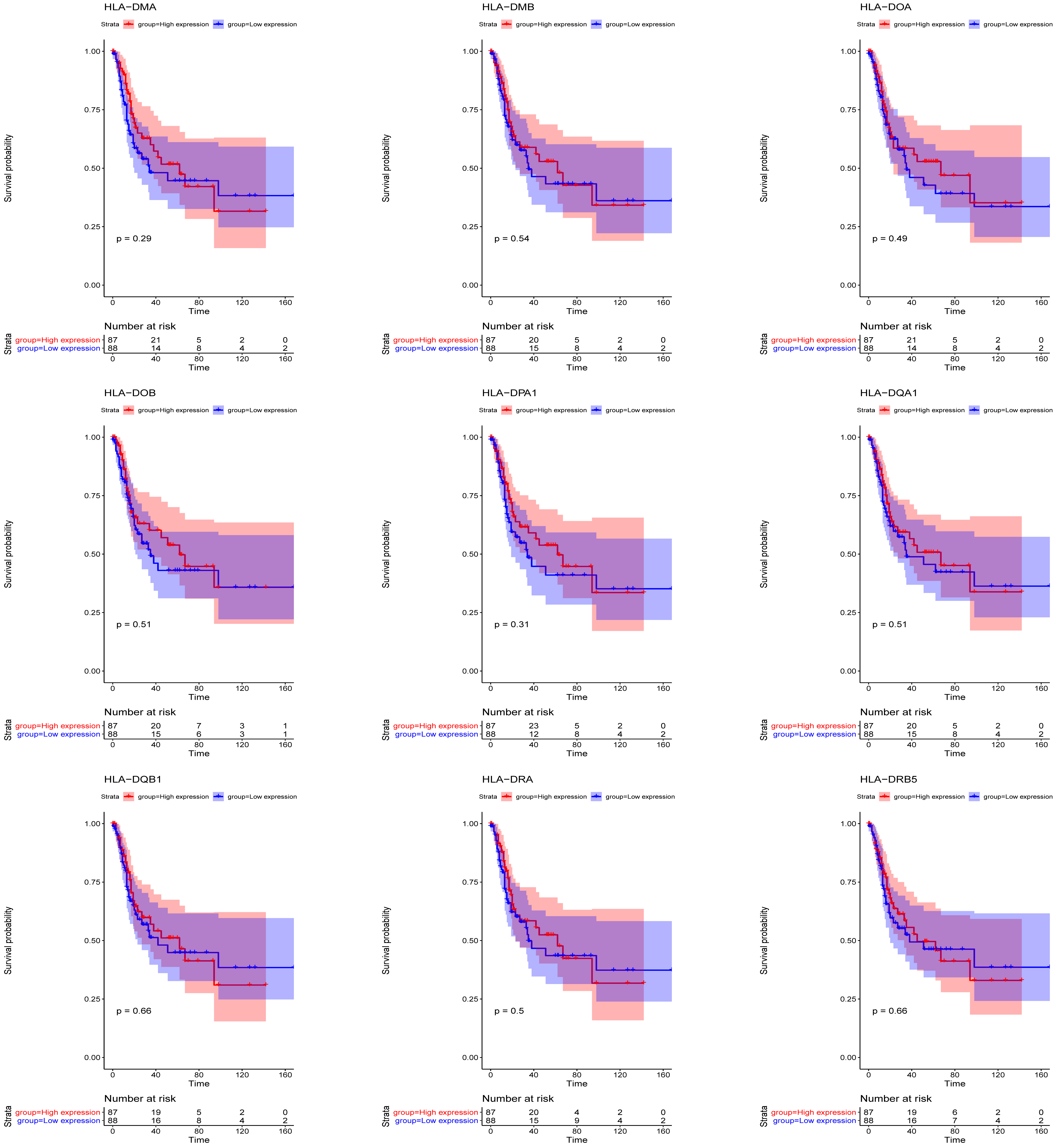

Supplement: Supplementary file 3 [file Image4.TIF]

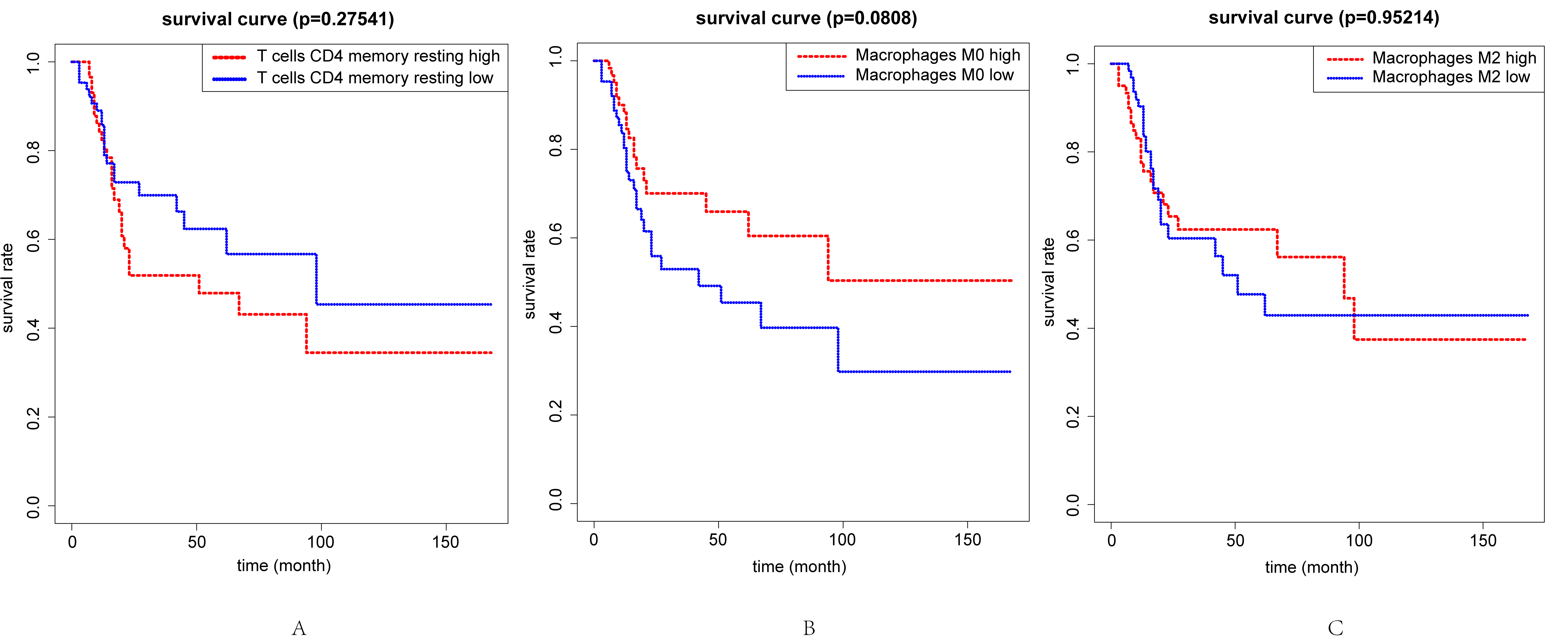

Supplement: Supplementary file 4 [file Image2.TIF]

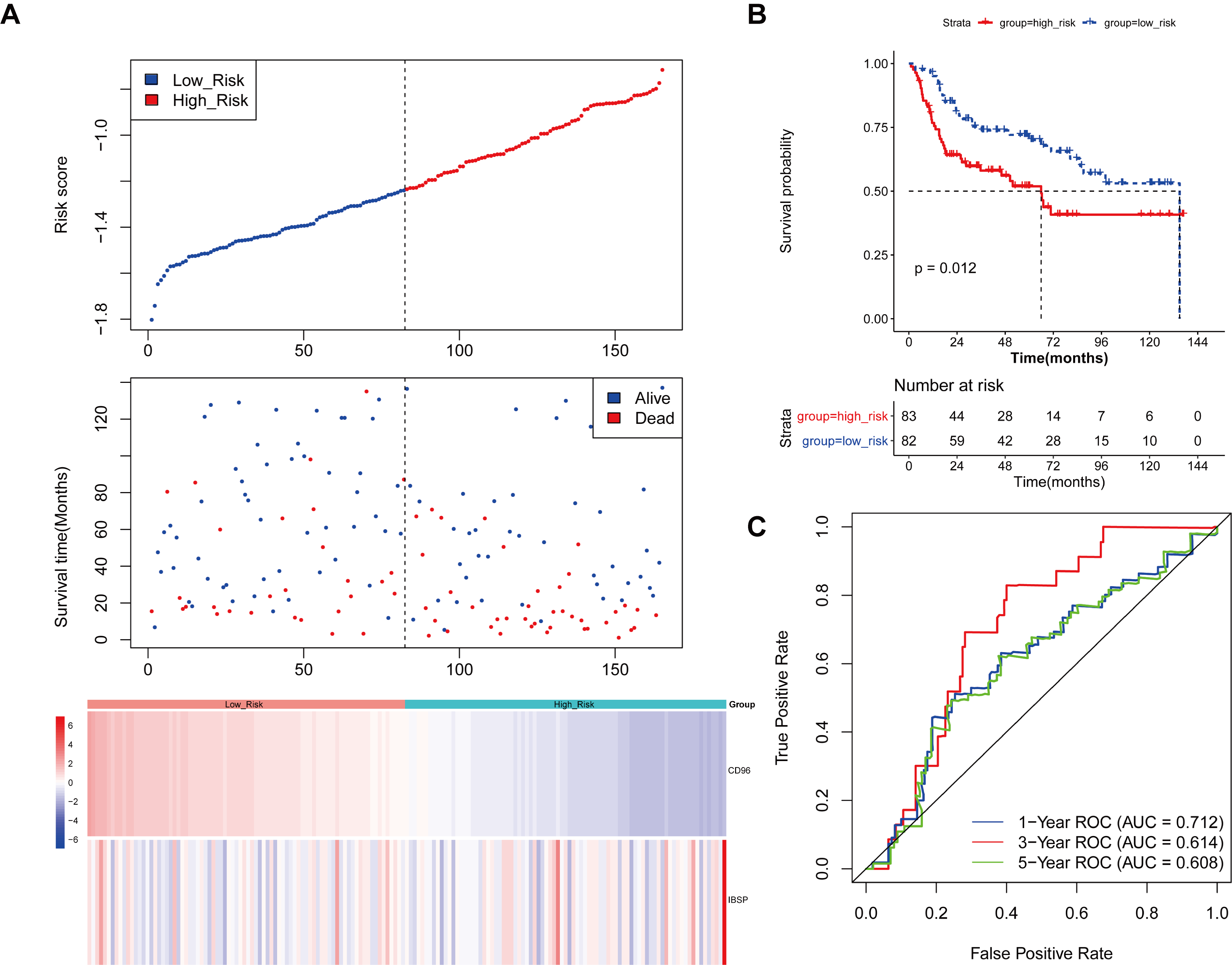

Supplement: Supplementary file 5 [file Image1.TIF]
