# Supplementary material for: New Prognostic Gene Signature and Immune Escape Mechanisms of Bladder Cancer
Source: Front Cell Dev Biol. 2022 May 12;10:775417. doi: 10.3389/fcell.2022.775417 (PMC9133907; doi:10.3389/fcell.2022.775417)
Supplement: Supplementary file 6 [file Table2.DOCX]

Supplementary Figure 1: A. Prognostic analysis of two-gene signature in the GSE13507 dataset. A. The curve of risk score (top). Survival status of the patients (middle). More dead patients correspond to the higher risk score. The dotted line represented the median risk score and divided the patients into low- and high-risk groups. Heatmap of the expression profiles of the two prognostic genes in low- and high-risk groups (bottom). B. Kaplan–Meier survival analysis of the two-gene signature. C. Time-dependent ROC analysis of the two-gene signature.

Supplementary Figure 2: Overall survival (OS) analysis of the three immune cells in BLCA based on the Kaplan-Meier plotter. The patients were stratified into high- and low- expression groups according to the median expression. (A) Resting memory CD4 T cells. (B) M0 macrophages. (C) M2 macrophages.

Supplementary Figure 3: Analysis of chemokines associated with the risk score. (A) Heat map of correlation between the risk score and differentially expressed chemokines (between ImmuneScoreH and ImmuneScoreL groups). The patients were stratified into high- and low- expression groups according to the median expression. (B1) CCL5. (B2) CCR5. (B3) CXCL9. (B4) CXCL13. (B5) CXCR3. (B6) CXCR6. (B7) XCL2.

Supplementary Figure 4: Prognostic analysis of nine differentially expressed antigen-presenting molecules (between ImmuneScoreH and ImmuneScoreL groups) associated with the risk score, including HLA-DOB, HLA-DOA, HLA-DMA, HLA-DRA, HLA-DMB, HLA-DQA1, HLA-DPA1, HLA-DQB1, and HLA-DRB5.

Supplementary Figure 5: Prognostic analysis of 15 differentially expressed immunomodulators (between ImmuneScoreH and ImmuneScoreL groups) associated with the risk score, including TIGIT.x, CD27, PDCD1, CD40LG, ICOS SLAMF7, CTLA4, CD28, BTLA, BTN3A1, CXCL9, CCL5, IL2RA, PRF1, and LAG3.
